# Spectroscopic Study of Five-Coordinated Thermal Treated Alumina Formation: FTIR and NMR Applying

**DOI:** 10.3390/ijms24065151

**Published:** 2023-03-08

**Authors:** Maxim Mashkovtsev, Nataliia Tarasova, Evgeniy Baksheev, Vladimir Rychkov, Nikolai Zhuravlev, Polina Solodovnikova, Maria Galiaskarova

**Affiliations:** 1 Institute of Physics and Technology, Ural Federal University, 620002 Yekaterinburg, Russia; 2 The Institute of High Temperature Electrochemistry of the Ural Branch of the Russian Academy of Sciences, 119991 Yekaterinburg, Russia; 3 Institute of Solid State Chemistry of the Ural Branch of the Russian Academy of Sciences, 119991 Yekaterinburg, Russia

**Keywords:** aluminum oxide, controlled double jet precipitation, penta-coordinated structure, nuclear magnetic resonance spectroscopy

## Abstract

This work represents research into materials designed to improve the environment. The study was carried out on aluminum hydroxide xerogels and alumina catalysts obtained by the Controlled Double Jet Precipitation (CDJP) process at different pH values. It has been shown that the pH of the CDJP process determines the content of aluminum-bound nitrate ions in the aluminum hydroxide. These ions are removed at a higher temperature than the decomposition of ammonium nitrate. The high content of aluminum-bound nitrate ions determines the structural disorder of the alumina and the high content of the penta-coordinated alumina catalyst.

## 1. Introduction

The quality of human life is improving with the development of materials in a wide range of applications. Improving the environment is essential to prevent many risks of various diseases, premature aging and industrial disasters. The demand for cost-effective and environmentally friendly materials to reduce waste management and pollution problems is increasing. Such inorganic materials as alumina can be used in the production of heterogeneous catalysts for oil refining processes [1,2,3], removal of fluorine from potable and wastewater [4], dehydrogenation of hydrocarbons [3,5], Claus process [6] and purification of exhaust gases from internal combustion engines. ɣ-Al_2_O_3_ is one of the metastable forms of aluminum oxide [7,8,9]. This form of alumina is characterized by a high thermally stable specific surface area and porosity [10,11,12]. 

The morphology and structure of ɣ-Al_2_O_3_ has a significant effect on its functional properties. In particular, unsaturated penta-coordinated Al^3+^ sites are of great interest for investigation due to structural instability and a tendency to coordination saturation. Thus, a strong interaction with dopants (mainly La or Zr) and catalytically active components (platinum group metals) is possible on penta-sites [13,14,15]. Due to this effect, the thermal stability of the surface area and the porous structure of the alumina-based support is significantly increased, which improves the catalytic properties. Many works have been devoted to studying the effect of Al^3+^ penta-sites concentration on the catalytic properties of alumina-based catalysts [16,17,18,19].

It is generally accepted that the main method of varying the concentration of penta-coordinated sites of alumina is to change the heat treatment conditions [17,18,19]. Heat treatment of aluminum hydroxide at temperatures up to 650 °C is accompanied by dehydration and hydroxylation processes that lead to the formation of coordinated unsaturated Al^3+^ penta-sites. These formed penta-sites are structurally unstable and tend to saturate coordinatively to hexa- and octa-forms with increasing temperature of heat treatment to 750 °C and above [17]. Wang et al. [20] found that the concentration of aluminum penta-sites can be controlled by using solvents with different enthalpies of combustion, resulting in different flame temperatures during aerosol decomposition. Interestingly, a higher flame temperature results in a sharper temperature drop during cooling, which contributes to the stabilization of the metastable aluminum penta-sites in the final product. 

Precipitation from aqueous solutions is the most common industrial method. The type of precursor and precipitant, as well as the precipitation conditions, affect the properties of the final product. Traditionally, there are three precipitation methods: variable pH (direct precipitation), constant pH in batch mode (reverse precipitation) and constant pH by controlled double jet precipitation (CDJP). The CDJP method allows the precipitation conditions to be reproducibly varied over a wide range and is considered the most promising method for producing alumina with the required properties.

It should be noted that a comprehensive study of the properties of aluminum hydroxide obtained under different precipitation conditions, and in particular the formation of coordinative unsaturated Al^3+^ sites, has not been carried out. Therefore, the aim of this work was to investigate the effect of the pH of the aluminum hydroxide precipitation during the CDJP process on the properties of xerogels and alumina. The properties of the aluminum hydroxide slurry and xerogels obtained after filtration and drying were studied in detail; in particular, the size and morphology of the particles as well as the chemical composition, which was determined by a complex of methods such as IR and TGA with mass spectrometry. The properties of the alumina obtained after the calcination of xerogels have also been studied in detail. In particular, the correlation between the precipitation pH of aluminum hydroxide and the concentration of penta-coordinated Al^3+^ sites were determined using NMR spectroscopy.

## 2. Results

### 2.1. Particle Size and Morphology Investigation during Precipitation

**Figure 1:**
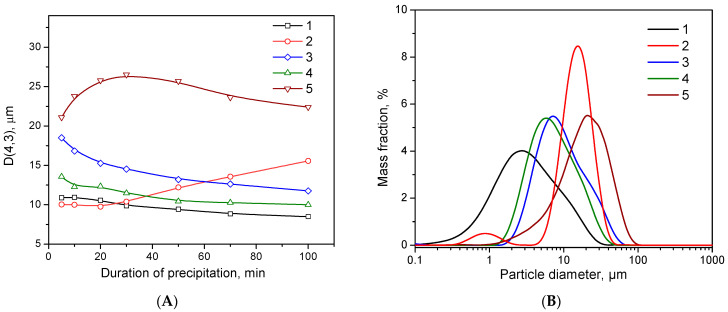
Dependence of the average particle diameter on the precipitation duration (**A**) and particle size distribution at the end of precipitation (**B**): (1) Al-5; (2) Al-6; (3) Al-7; (4) Al-8 and (5) Al-9.

By the 20th minute of the CDJP process, the hydroxide slurries of Al-5 and Al-6 were characterized by low viscosity, whereas viscous gel-like slurries were formed in the case of samples Al-7, Al-8 and Al-9. Figure 1A shows the changes in mean particle diameter during the precipitation process. Interestingly, in the initial stages of precipitation, the smallest particle size was observed at the level of 10 μm for samples Al-5 and Al-6. While the particle diameter of the Al-5 sample remains almost constant throughout the precipitation, the particle diameter of the Al-6 sample systematically increases from 30 min of CDJP. The average particle diameter decrease during the CDJP process was observed for Al-7 and Al-8 samples. The largest average particle diameter was characterized for Al-9. Particle size distributions for all samples at the end of precipitation are shown in Figure 1B. A bimodal particle size distribution with a narrow main peak in the 15 µm region and a small peak in the 0.9 µm region was observed for Al-6. Broad monomodal distributions with shoulders either in the large size range (samples Al-5, Al-7 and Al-8) or in the small size range (sample Al-9) were found.

**Figure 2:**
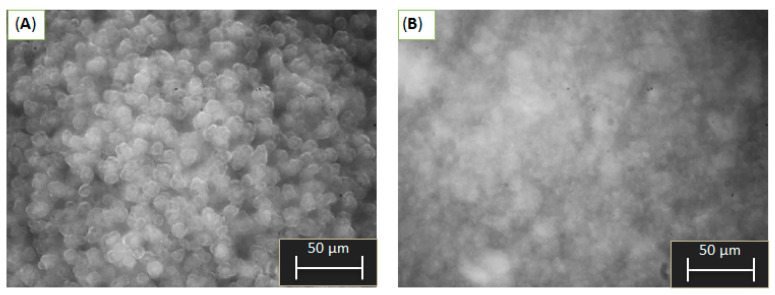
Optical image of suspension particles at the end of precipitation Al-6 (**A**) and Al-9 (**B**).

Figure 2 shows optical images of Al-6 and Al-9 slurry particles. Other samples are shown in the Supplementary Materials (Figure S2). The particles of the Al-6 sample have clear boundaries and a near-spherical shape. Optical microscopy and laser diffraction data are in good agreement with respect to particle size. From Figure 2B it can be seen that Al-9 consists of irregularly shaped flocs with blurred boundaries and a wide range of particle sizes. The shape and particle size of the other samples could not be distinguished by optical microscopy. Figure S1 shows particle size distribution curves for different CDJP durations for Al-6. Interestingly, the main peak systematically shifts to the larger size region during the precipitation process, indicating an increase in the particle size of the main population. This is probably caused by layer-by-layer primary particle growth of aluminum hydroxide (a small peak in the region of 0.9 μm) on the surface of spheroidal Ag aggregates.

Therefore, the pH of the CDJP process has a significant effect on the particle size and morphology of the aluminum hydroxide. Well-sedimented spherical particles with a narrow particle size distribution and well-defined boundaries are formed only at pH = 6, while flocculent particles are formed at all other precipitation pHs.

### 2.2. Xerogels Composition and Structure

**Figure 3:**
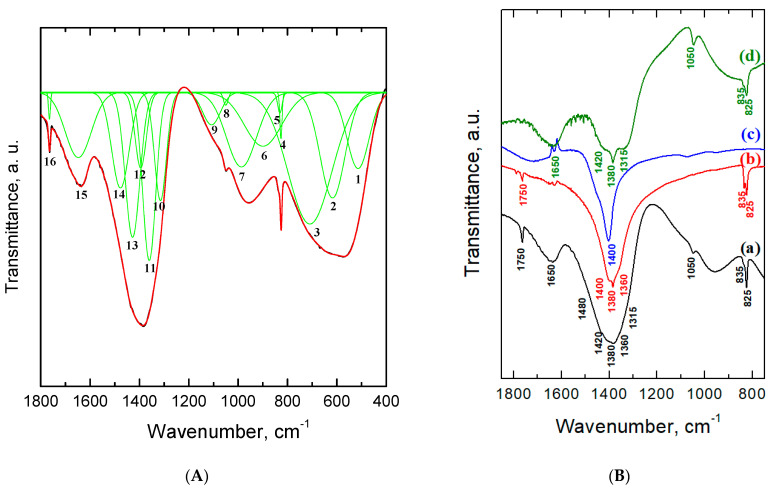
Deconvolution (green line) of the IR spectrum (red line) of the Al-5 sample (**A**) and comparison of the IR spectrum with precursors (**B**): (a) Al-5; (b) NH_4_NO_3_; (c) NH_4_Cl and (d) Al(NO_3_)_3_∙9H_2_O.

Xerogels were obtained after filtering and drying the precipitates after the CDJP process. It was shown that the Al-5 and Al-6 xerogels were friable powders, whereas the Al-7–Al-9 samples formed dense fragmented aggregates during drying. Optical images of the xerogels are shown in the Supplementary Material (Figure S3). The local structural analysis of the xerogels was carried out using the IR spectroscopy method. Deconvolution was performed on the spectrum of the Al-5 sample (Figure 3A). The assignment of each signal to a particular vibrational mode is shown in Table 1. Figure 3B compares the spectrum of Al-5 (curve a) with the spectra of ammonium nitrate (curve b), ammonium chloride (curve c) and aluminum nitrate (curve d).

The ν_1_, ν_2_ and ν_3_ signals recorded in the low-frequency region of the Al-5 sample spectrum correspond to stretching vibrations of the Al–O aluminum–oxygen bond [21]. The ν_4_ signal can be attributed both to the same vibrations and to bending vibrations of the O–N–O bond in nitro groups [22]. Signals ν_7_, ν_8_ and ν_10_ characterize bending vibrations of the Al–OH bond [23,24]. The ν_5_, ν_6_ and ν_14_ signals are present both in the Al-5 spectrum and in the spectra of ammonium nitrate and aluminum nitrate, which makes it possible to unambiguously attribute these signals to the vibrations of nitro groups. The ν_13_ and ν_19_ signals are present in the spectra of Al-5 and NH_4_NO_3_, while the ν_9_ and ν_15_ signals are present in the spectra of Al-5 and Al(NO_3_)_3_∙9H_2_O, which also makes it possible to identify these signals as vibrational signals of nitro groups. The ν_18_ signal recorded both in the spectra of Al-5 and Al(NO_3_)_3_∙9H_2_O corresponds to vibrations of the H_2_O water molecules contained in the structure of these compounds [25]. The ν_19_ signal observed in the spectra of Al-5 and NH_4_NO_3_ characterizes the stretching vibrations of ammonium cations and nitrate ions, which are simultaneously present in the samples [26]. This suggests that the nitro groups in these samples are bound both to aluminum and to ammonium cations.

One can expect the appearance of four signals with frequencies of 719 cm^–1^ (in-plane deformation vibration), 730 cm^–1^ (out-of-plane rocking vibration), 1050 cm^–1^ (symmetric N–O stretches) and 1380 cm^–1^ (asymmetric N–O stretches) for free nitro groups in the spectrum [27]. Low-frequency region analysis of vibrations (below 800 cm^–1^) is complicated by the superposition of the metal–oxygen stretching vibrations bonds signals and bending vibrations of nitrogen–oxygen bonds. Therefore, the ν_4_ signal cannot be unambiguously attributed to a certain type of vibration. The doublet ν_5_ and ν_6_ can be attributed to out-of-plane rocking vibrations of N–O bonds. The ν_9_ and ν_14_ signals can be attributed to symmetric and asymmetric vibrations of N–O bonds in free nitrate ions, respectively. At the same time, the ν_9_ signal is recorded in the spectra of the Al-5 sample and aluminum nitrate and absent in the spectra of ammonium nitrate and ammonium chloride, which can also belong to the bending vibrations of the Al–O–NO_2_ bonds. For the nitro groups associated with ammonium or metal cations, a decrease in symmetry is observed, which leads to an increase in the number of signals in the IR spectra. 

Thus, the number of signals can increase to six or more for metal-coordinated nitrate ions. This is due to the appearance of differently coordinated nitro groups, such as monodentate and bridging ones [28]. The presence of monodentate nitro groups in the spectrum of the Al-5 sample is confirmed by the presence of the ν_11_ signal corresponding to symmetrical vibrations of N–O bonds in nitro groups of this type [22]. The complex signal in the frequency range 1300–1500 cm^–1^ is a superposition of the ν_12_, ν_13_, ν_14_, ν_15_, ν_16_ and ν_17_ signals characterizing asymmetric vibrations of N–O bonds in nitro groups (Figure S4). The ν_13_ and ν_15_ signals can characterize N–O vibrations in monodentate nitro groups, while the ν_12_, ν_16_ and ν_17_ signals can do so in the bridging ones [28]. The simultaneous presence of signals inherent in Al–O–NO_2_ bonds in the spectra, monodentate and bridging nitro groups indicates the presence of nitrate ions bound directly to aluminum.

Figure S5 demonstrates the IR spectra for all xerogel samples. It was shown that the complex signal in the region of 1300–1500 cm^−1^ narrows as the pH value of the CDJP process increases due to a decrease in the relative intensity of the ν_12_, ν_16_ and ν_17_ components. Furthermore, the intensity of this complex signal increases relative to the intensity of other signals associated with vibrations of the metal sub-lattice Al–O (ν_1_, ν_2_, ν_3_), Al–OH (ν_7_, ν_8_, ν_10_) and water in crystallization form (ν_18_). Thus, nitrate ions are contained in all samples, both associated with ammonium and directly with aluminum, and with an increase in precipitation pH the total content of nitrate ions in the samples increases.

### 2.3. Chemical Composition of Xerogels

The data in Table 2 show that as the pH of the CDJP process increases, the alumina content of the xerogels decreases due to an increase in salts and water content. At the same time, as the pH increases from 5 to 8, the content of salts in the xerogels systematically increases and the water content decreases. On the other hand, at pH 8 to 9, the salt content decreases and the water content increases. Figure S6 shows the content of components in xerogels at different pH values. The lowest content of water and salts is typical for xerogels obtained from well-sedimented suspensions. Interestingly, the amount of ammonium ions is significantly lower than the number of nitrate ions in all xerogels. This confirms the IR spectroscopy data on the presence of nitrate ions directly coordinated with aluminum. This was used to estimate the number of nitrate ions associated with ammonium and aluminum. It was found that as the pH of the CDJP process increases, the content of ammonium-bound nitrate ions in xerogels decreases from 17 to 4 wt%, which corresponds to a decrease in the NO_3_^−^*/Al molar ratio from 0.35 to 0.15. Thus, during precipitation over a wide range of pH values, the OH groups of the aluminum hydroxide are partially replaced by nitrate ions, and this replacement is most pronounced at low pH values. Probably due to the large number of nitrate ions in the mother liquor, as well as the local decrease in pH at the point of aluminum nitrate solution drop; the formation of a basic salt occurs during the hydrolysis process.

**Figure 4:**
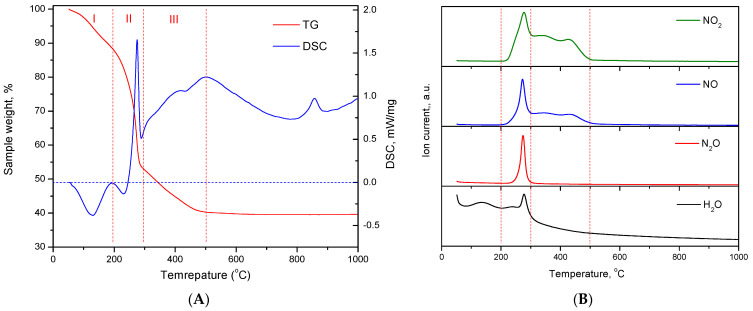
Thermal analysis (**A**) and mass-ion curves (**B**) of the Al-5 sample.

Thermal analysis was carried out to confirm the chemical composition of the xerogels. Figure 4A shows the thermogram of Al-5 and the differential thermal analysis curve. Figure 4B shows the gas phase mass ion curves. It has been shown that the decomposition of the xerogel proceeds in three stages. In the first stage, in the temperature range from 25 °C to 200 °C, water is removed, accompanied by an endothermic effect and a peak on the mass ion curve at mass number 17 (OH groups). From 200 °C to 300 °C, ammonium nitrate decomposes, accompanied by an intense exothermic effect and peaks of N_2_O, NO and NO_2_ release. The release of N_2_O indicates the decomposition of the ammonium group. In the third stage, from 300 °C to 500 °C, the decomposition of nitrate ions associated with aluminum takes place. In this case, there are no thermal effects and only NO and NO_2_ are released into the gas phase. Weight loss curves for samples obtained at different pH are shown in the Supplementary Materials (Figure S7) and a summary of weight loss at each stage is given in Table 3.

**Figure 5:**
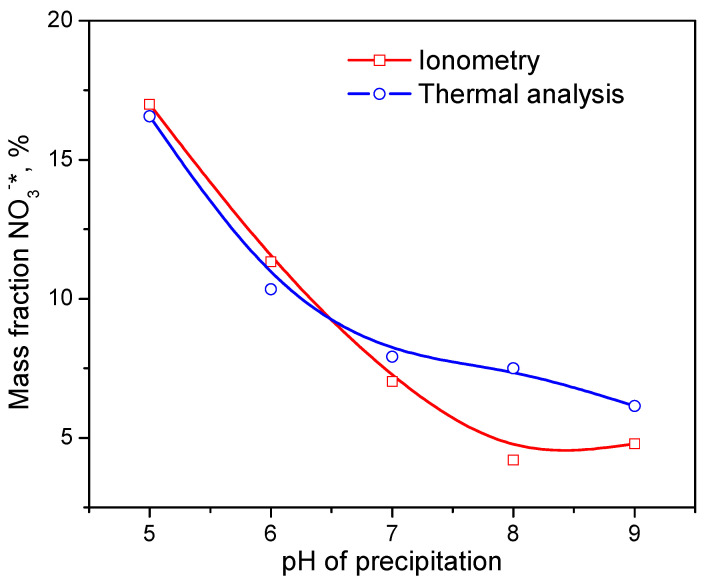
The content of aluminum-bound nitrate ions NO_3_^−^* (not included in NH_4_NO_3_) according to ionometry and thermal analysis.

The total weight loss of the samples and the content of nitrate ions associated with aluminum are generally in good agreement with the ionometric data. Figure 5 shows the dependence of the NO^3−^*content on the pH of the CDJP process determined by different methods. It should be noted that the peak of water removal from xerogels is very broad and is superimposed on both the peak of ammonium nitrate decomposition and the peak of removal of aluminum-bound nitrate ions. As a result, the water content in xerogels obtained by thermal analysis is significantly lower than the ionometric data, and the content of ammonium nitrate is significantly higher. In general, we consider the results of ionometry to be more reliable due to the overlap of peaks in thermal analysis.

It was found that aluminum hydroxide xerogels contain water, ammonium nitrate and nitrate ions directly bound to aluminum. The content of aluminum-coordinated nitrate ions, as well as the NO_3_^−^*/Al molar ratio, decreases as the pH of the CDJP process increases. According to thermal analysis, the removal of nitrate ions from the samples is complete at 500 °C.

### 2.4. Aluminum Oxide Samples Research

**Figure 6:**
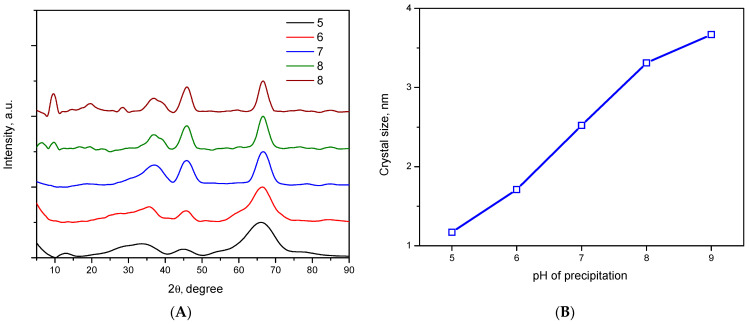
X-ray patterns of the samples (**A**): (5) Al-5; (6) Al-6; (7) Al-7; (8) Al-8; (9) Al-9 and the dependence of the crystal size on the pH of the precipitation (**B**).

In this work, the calcination temperature of the xerogels was 500 °C. At this temperature the formation of low temperature γ, η, χ, ρ and x metastable forms of alumina occur. Figure 6A shows that all samples were characterized by a small crystallite size, resulting in a large width of the diffraction peaks, making it difficult to accurately determine the phase composition of the alumina samples. However, the position of the diffraction peaks on the X-ray diffraction patterns for all alumina samples is well described by the γ-phase, which is characterized by a pseudo-amorphous structure of defective spinel in which Al^3+^ cations and vacancies are randomly distributed between tetrahedral and octahedral positions in the perfect FCC sublattice of oxygen atoms, which is in agreement with crystallographic data [29]. In addition, the low crystallinity of the samples can be related to the presence of a small amount of structurally bound water, which remains until the phase transition to the stable α-phase of alumina. As the precipitation pH increases, a decrease in peak width at half height is observed, indicating an increase in crystallite size. Figure 6B shows that the crystallite size increases almost linearly with the pH growth of the CDJP process. It is possible that the high content of nitrate ions associated with aluminum, which are removed at high temperature, prevents the ordering of the aluminum oxide.

**Figure 7:**
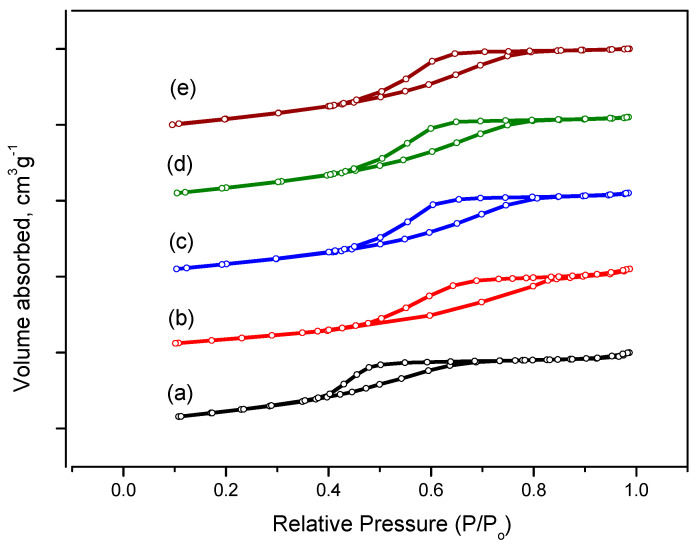
Nitrogen adsorption-desorption isotherm curves for fresh Al_2_O_3_ samples prepared at various pH values: (a) Al-5; (b) Al-6; (c) Al-7; (d) Al-8 and (e) Al-9.

**Figure 8:**
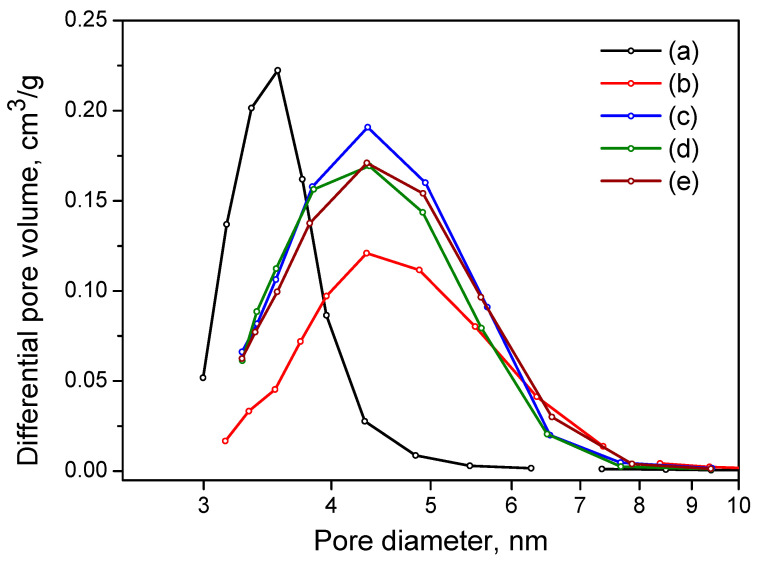
Pore size distribution curves for fresh Al_2_O_3_ samples prepared at various pH values: (a) Al-5; (b) Al-6; (c) Al-7; (d) Al-8 and (e) Al-9.

The N_2_ adsorption isotherms (Figure 7) for all samples correspond to type IV with the presence of the hysteresis loop of IUPAC type H2, indicating the predominance of ink bottle pores. For the samples obtained at pH = 6–9, the adsorption and desorption isotherms are practically indistinguishable, indicating the similarity of pore size and volume. For the pH = 5 sample, the hysteresis loop is observed at lower relative pressures, indicating the formation of smaller pores in the sample. This is clearly seen in the pore size distributions (Figure 8). The sample obtained at pH = 5 has a narrow pore size distribution ranging from 3 to 5 nm with a peak at 3.5 nm. Samples prepared at pH = 6–9 show a peak shift to around 4.3 nm and a wider pore size distribution ranging from 3 to 8 nm.

In general (Table 4), an increase in both the specific surface area and pore volume of the samples is observed with the pH growth of the CDJP process. For samples obtained from sedimentation suspensions, the specific surface area and pore volume are at the level of 200 m^2^/g and 0.28 mL/g, respectively. The specific surface and pore volume of the samples obtained from gel suspensions are at the level of 230 m^2^/g and 0.4 mL/g, respectively.

**Figure 9:**
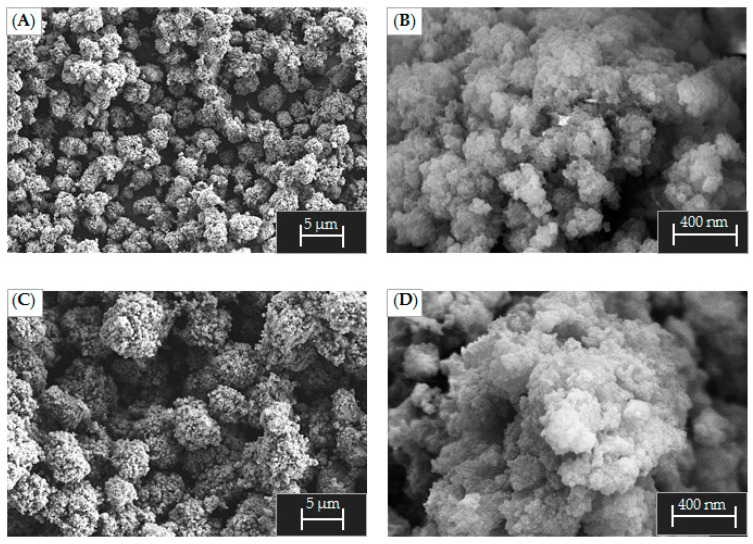
SEM images for Al-5 and Al-6 samples: (**A**) Al-5 magnification 1k; (**B**) Al-5 magnification 50k; (**C**) Al-6 magnification 2.5k; (**D**) Al-6 magnification 50k.

The particles of the alumina sample obtained at pH 5 (Figure 9A,B) are loose spherical agglomerates with a size of 5 to 10 μm consisting of primary porous particles with a size of 30 to 50 nm. The morphology of Al-6 (Figure 9C,D) is similar to that of Al-5. The agglomerates are 10 to 20 µm in size and consist of more tightly bound primary particles.

Figure S8 shows SEM images of Al-7, Al-8 and Al-9 samples after aging at 500 °C. The samples contain large fragments and small particles. The surface of the Al-7 sample has a porous and loose structure. For Al-8, a denser surface consisting of smaller particle aggregates was observed. Al-9 particles are large and fragmented aggregates with a loose surface consisting of densely agglomerated primary particles.

### 2.5. NMR-Spectum Research

**Figure 10:**
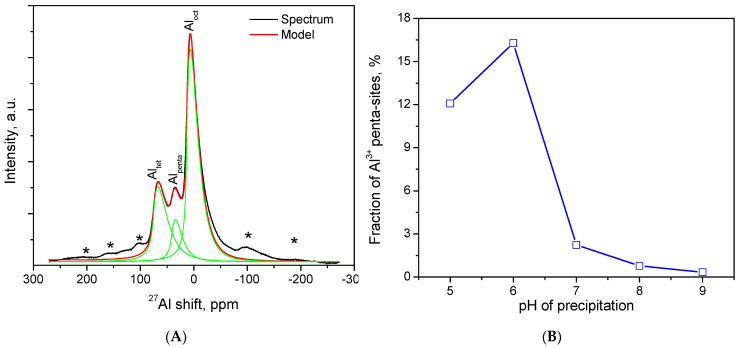
Deconvolution (green line) of the NMR spectrum (fitting—red line, experimental data—black line) for Al-5 sample, where asterisked (*) signals refer to rotational wiggles (**A**) and dependence of penta-sites of alumina on the precipitation pH (**B**).

The ^27^Al NMR spectra for Al-5 are shown in Figure 10A. It can be seen that three characteristic peaks at 10, 45 and 74 ppm chemical shift can be observed, which are assigned to Al^3+^ ions with octahedral, pentahedral and tetrahedral coordination environments, respectively [13,19]. The asterisked signals refer to rotational wiggles (MAS). Al-5 is rich in penta-coordinated Al^3+^ sites, and the integration of the peak areas of the aluminum sites in different coordination environments indicates that the fraction of penta-coordinated Al^3+^ sites is 12 at. %. It has been shown (Figure 10B) that in Al-5 and Al-6 samples obtained from well-sedimented suspensions, the content of penta-coordinated atoms is at the level of 12 to 17 at. % and significantly exceeds the content of such atoms in samples obtained from gel-like suspensions. Thus, as the pH of the CDJP process increases, a systematic decrease in the content of penta-coordinated Al^3+^ sites in the samples is observed. The content of penta-coordinated Al^3+^ sites correlates well with the content of aluminum-bound nitrate ions in xerogels, suggesting that these ions are involved in the formation of penta-coordinated centers during the heat treatment of aluminum hydroxide.

The content of penta-coordinate Al^3+^ centers in aluminum oxide samples correlates well with the content of aluminum-bound nitrate ions in aluminum hydroxide xerogels. It is likely that aluminum-bound nitrate ions play an active role in the formation of penta-coordinate centers during the heat treatment of aluminum hydroxide, mainly due to their increased thermal stability. At the same time, it has been reliably shown that varying the precipitation pH is an effective way of controlling the content of aluminum-bound anions. In turn, further study of the mechanism of the influence of aluminum-bound anions on the formation of pentacoordinate centers during the thermal decomposition of aluminum hydroxide is of great importance. It is possible to obtain alumina with a controlled content of pentacoordinate centers. This can be used to improve modern catalyst supports and adsorbents.

## 3. Materials and Methods

### 3.1. Synthesis of Aluminum Oxide

Synthesis of the samples was carried out as follows: aluminum nitrate solution and aqueous ammonia were fed drop-wise so that the pH value was constant during the precipitation process (∆pH ± 0.1 units). Several parallel syntheses were carried out at a pH value of 5, 6, 7, 8 and 9. Solutions with a concentration of C(Al^3+^) = 1 M and 10 wt.% ammonia were chosen for precipitation. The temperature in the reactor was 25 °C, the stirrer rotation speed was 500 rpm and the aluminum salt solution feed rate was 10 mL/min. After the precipitation was completed, the precipitate was separated from the mother liquor on a vacuum suction filter; then, the samples were dried at room temperature for 24 h and in an oven for 4 h at a temperature of 130 °C; then, the samples were fired for 4 h at a temperature of 500 °C. The samples are designated Al-5, Al-6, Al-7, Al-8 and Al-9, respectively.

### 3.2. Research Methods

Particle size distribution of the aluminum hydroxide slurry was characterized by laser diffraction with the Analysette 22 NanoTecPlus analyzer (Fritsch). Optical images were captured using an Olympus GX-71 microscope. FT-IR spectra of xerogels were recorded on a Vertex 70 infrared spectrometer (Brucker) using the Fourier transform in the wave number range of 500–3700 cm^−1^ with a step of 2 cm^−1^. The chemical composition of the xerogels was determined by the ion exchange method. To do this, 1 g of xerogel and 50 mL of MgSO_4_ solution with a concentration of 0.1 M were mixed in centrifuge tubes, shaken and subjected to ultrasonication to shatter aggregates, after which they were stirred for 24 h. After the sedimentation of the particles, the content of nitrate ions and ammonium ions in the liquid part of the suspension was determined using appropriate ion-selective electrodes. Three parallel measurements were carried out to determine the standard deviation. Thus, the standard deviation of the content of NH^4+^ and NO^3–^ ions in the xerogel did not exceed 10 and 5 wt %, respectively. Thermal analysis was carried out on an STA 449 F1 Jupiter (Netzsch) in platinum crucibles followed by the analysis of leaving gases on an Aeolos QMS 403 C (Netzsch) mass spectrometer. The heating rate was 10 °C/min and the temperature range was 25–1000 °C. 

Analysis of the surface morphology and particle size of the alumina samples were observed from microphotographs using an AURIGA CrossBeam scanning electron microscope (Carl Zeiss Group). Surface area and porosity were estimated by processing the isotherms of low-temperature adsorption-desorption of nitrogen on a Nova Series 1200e analyzer (Quantachrome Instruments) by the BET and BJH methods. The X-ray phase analysis was carried out on an X-Pert Pro MRD X-ray diffractometer (Panalytical). ^27^Al NMR measurements were performed on an Agilent 400WB magnet (B0 = 9.4 T and ^27^Al Larmor frequency of 104.31 MHz) with magic-angle spinning (MAS) at 10 kHz using a 4 mm zirconia rotor. The ^27^Al spectra were referenced to 1.1 M Al(NO_3_)_3_. Accurate intensities of the contributions to each spectrum were obtained through a fitting of the resonance line shapes with Czjzek distribution using the DMFIT program [30,31,32].

## 4. Conclusions

It was found that the pH of the CDJP process has a significant effect on the content of penta-coordinated aluminum atoms. In this case, it is generally observed that the content of penta-sites decreases as the pH of the CDJP process increases. At low pH, aluminum hydroxide with a high content of nitrate ions unbound to ammonium is formed. Presumably, a local pH decrease at the point of introduction of the aluminum nitrate solution into the reaction volume causes the replacement of some of the hydroxyl groups by nitrate ions. The lower the pH of the reaction volume, the greater the local shortage of hydroxide ions which, in combination with a high ammonium nitrate content in the reaction volume, leads to the formation of basic aluminum nitrate. Nitrate ions associated with aluminum are more thermally stable than nitrate ions associated with ammonium. Their removal from the aluminum hydroxide occurs in the range of 280 to 500 °C. The high content of these ions in the precipitated slurry samples leads to the formation of disordered alumina with small crystallite size during thermal treatment. In turn, the high disorder is consistent with a high content of penta-coordinated alumina. A decrease in the content of nitrate ions associated with aluminum leads to an increase in the size of the crystallites and a decrease in the content of penta-sites. It should be noted that in this case, the specific surface area of the Al^3+^ alumina is only slightly dependent on the pH of the CDJP process, which makes it possible to use the synthesized alumina samples as catalyst supports.

## Figures and Tables

**Table 1:** Wavenumbers of IR bands of Al-5 composition.

No of Band	Wavenumber, cm^–1^	Description
1	480	ν(Al–O)
2	535	ν(Al–O)
3	625	ν(Al–O)
4	725	ν(Al–O) or δ(O–N–O)
5	825	out-of-plane rocking vibration of N–O
6	835	out-of-plane rocking vibration of N–O
7	900	δ(Al–OH)
8	985	δ(Al–OH)
9	1050	δ(Al–O–NO2) or ν_s_(N–O)
10	1100	δ(Al–OH)
11	1275	ν_s_(N–O)
12	1315	ν_as_(N–O)
13	1360	ν_as_(N–O)
14	1380	ν_as_(N–O)
15	1420	ν_as_(N–O)
16	1480	ν_as_(N–O)
17	1520	ν_as_(N–O)
18	1650	vibration of H_2_O molecules
19	1750	stretches vibration of NH_4_^+^ + NO_3_^–^

**Table 2:** Chemical composition of xerogels.

Sample	Mass Fraction, %				Mole Ratio			
	Al_2_O_3_	NH_4_^+^	NO_3_^–^	H_2_O	NH_4_^+^/Al	NO_3_^–^/Al	NO_3_^–^*/Al	H_2_O/Al
Al-5	39.6	3.1	27.8	29.4	0.22	0.58	0.35	2.10
Al-6	33.1	7.4	36.9	22.6	0.63	0.92	0.28	1.93
Al-7	25.9	10.5	43.3	20.2	1.15	1.37	0.22	2.21
Al-8	23.7	12.7	47.9	15.7	1.52	1.66	0.15	1.88
Al-9	21.5	9.2	36.4	32.9	1.21	1.39	0.18	4.34

NO_3_^−^*—Nitrate ions not included in NH_4_NO_3_.

**Table 3:** Results of thermal analysis.

Sample	Peak 1		Peak 2		Peak 3		Total Weight Loss, %
	T, °C	Δm, %	T, °C	Δm, %	T, °C	Δm, %	
Al-5	35–200	13.63	200–279	30.55	279–600	16.56	60.74
Al-6	35–200	12.43	200–289	44.61	289–600	10.34	67.38
Al-7	35–200	7.87	200–302	58.53	302–600	7.92	74.32
Al-8	35–200	5.8	200–303	61.53	303–600	7.5	74.83
Al-9	35-200	5.63	200-308	66.47	308-600	6.14	78.24

**Table 4:** Textural properties of Al_2_O_3_ samples prepared at various pH values.

Samples	Surface Area, m^2^/g	Pore Volume., cm^3^/g	Pore Size., nm
Al-5	206	0.23	3.5
Al-6	200	0.33	4.3
Al-7	210	0.43	4.3
Al-8	240	0.38	4.3
Al-9	236	0.40	4.3

## Data Availability

Not applicable.

## Supplementary Materials

The following supporting information can be downloaded at: https://www.mdpi.com/article/10.3390/ijms24065151/s1.
