# Supplementary material for: Spectroscopic Study of Five-Coordinated Thermal Treated Alumina Formation: FTIR and NMR Applying
Source: Int J Mol Sci. 2023 Mar 8;24(6):5151. doi: 10.3390/ijms24065151 (PMC10049124; doi:10.3390/ijms24065151)
Supplement: Supplementary file 1 [file ijms-24-05151-s001.zip › ijms-2249351-supplementary.pdf]

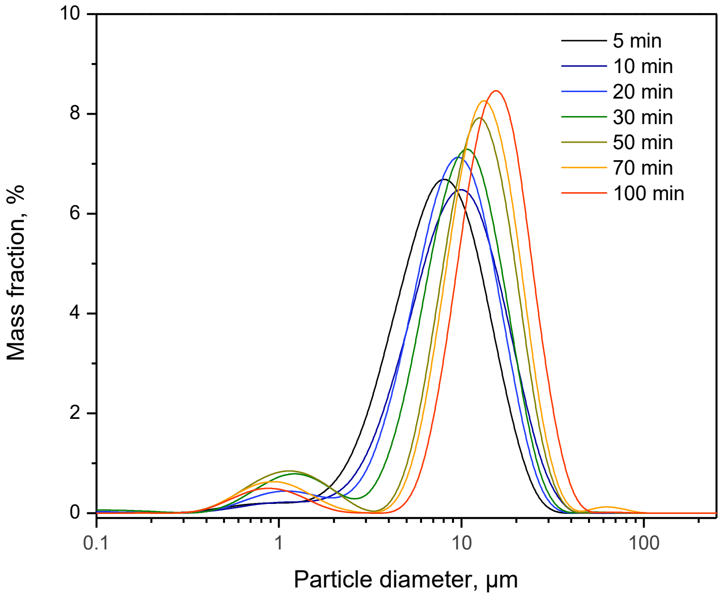

Figure S1. Particle size distribution curves at different CDJP stages of Al-6.

Figure S1 shows particle size distribution curves with various duration of CDJP for Al-6. It was shown that the main peak systematically shifts to the region of large sizes during the process of precipitation.

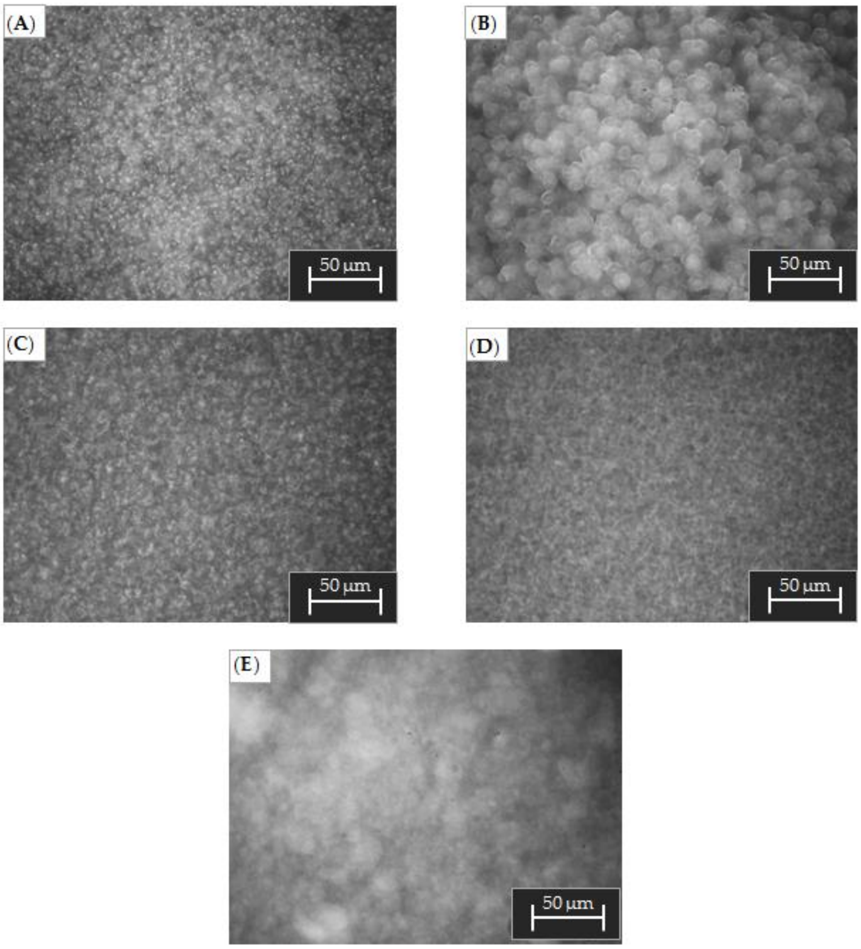

Figure S2. Optical image of suspension particles at the end of deposition: (**A**) Al-5; (**B**) Al-6; (**C**) Al-7; (**D**) Al-8 and (**E**) Al-9.

The precipitates of Al-5 and Al-6 were characterized by spherical shape particles, with Al-6 particles being larger and having clear boundaries. Samples of Al-6 and Al-7 contain small irregular shape particles, and the Al-9 sample was characterized by amorphous gel morphology.

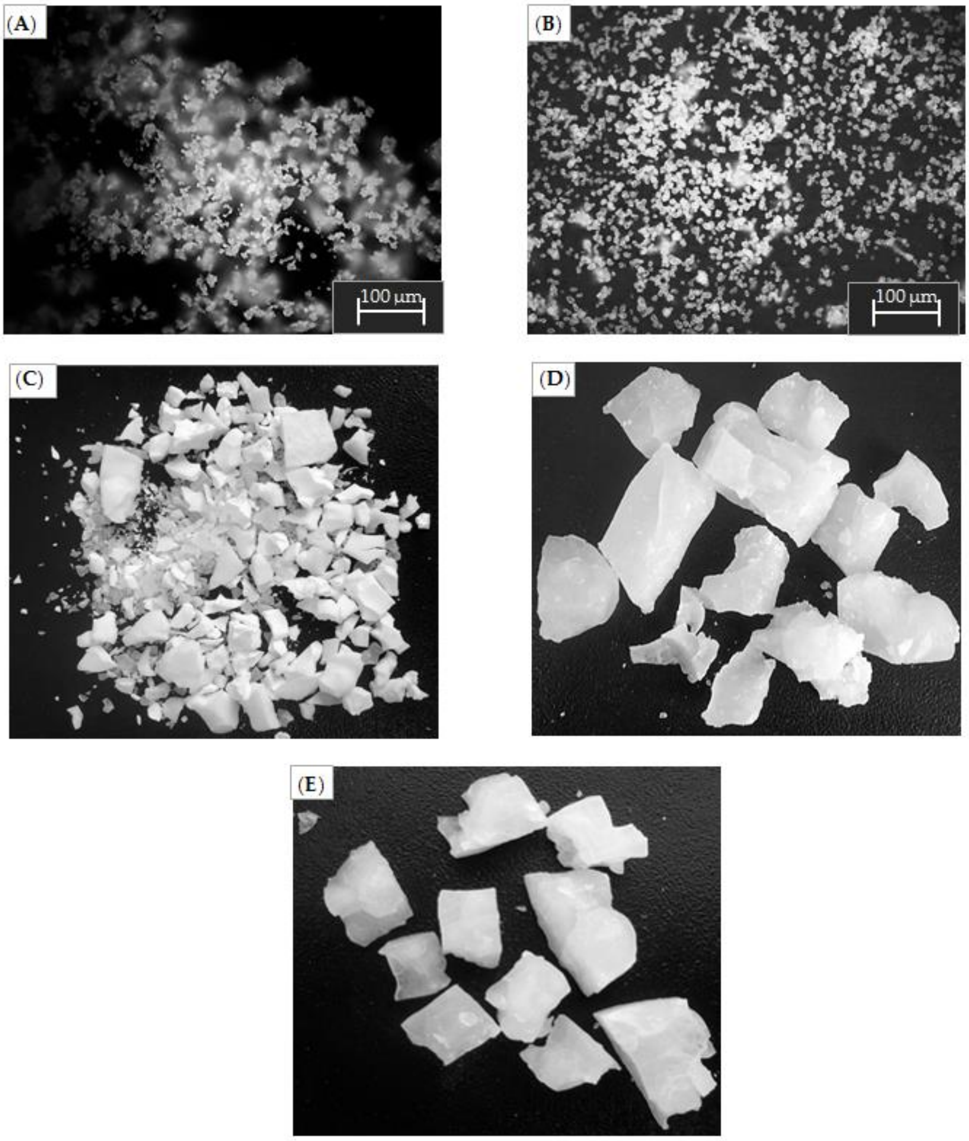

Figure S3. Optical images (**A**,**B**) and photos (**C**–**E**) of xerogels after drying: (**A**) Al-5; (**B**) Al-6; (**C**) Al-7; (**D**) Al-8 and (**E**) Al-9.

A strong difference was observed between the dried hydroxides. Samples of Al-5 and Al-6 are well crumbling powder. Conversely, Al-7, Al-8 and Al-9 specimens form translucent large fragments.

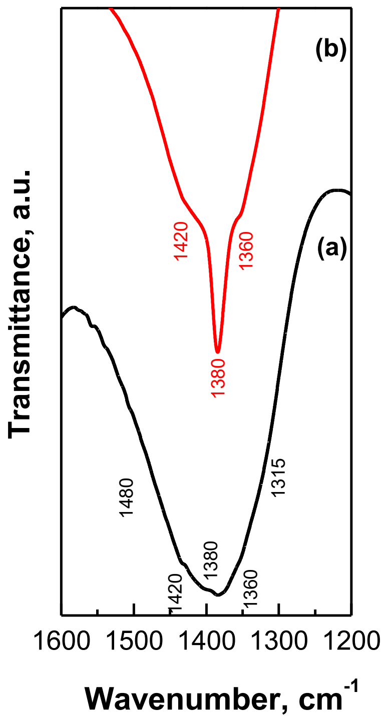

Figure S4. Fragment of IR spectra for xerogels: (a) Al-5 and (b) Al-9.

Figure S4 shows the fragment of the IR spectrum in the region of 1300–1500 cm^{-1}, in which the signals correspond to vibrations of the N–O bond. For Al-9 a significant narrowing of the signal intensity was observed.

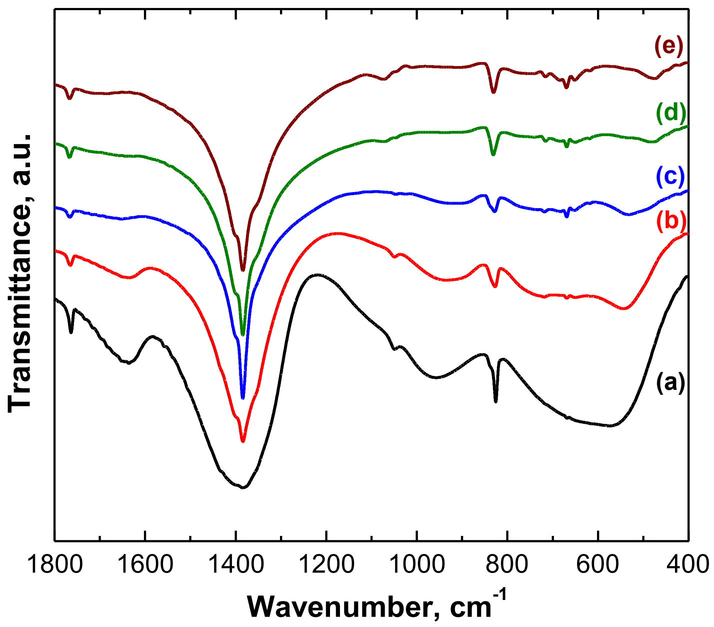

Figure S5. IR spectra of xerogels: (a) Al-5; (b) Al-6; (c) Al-7; (d) Al-8 and (e) Al-9.

Figure S5 demonstrates the IR spectra for all samples of xerogels. It was shown that the complex signal in the region of 1300–1500 cm^{-1} narrows as the CDJP process pH value increases. Furthermore, the

intensity of this complex signal increases relative to the intensity of other signals associated with vibrations of the metal sub-lattice Al–O, Al–OH and the water in crystallization form.

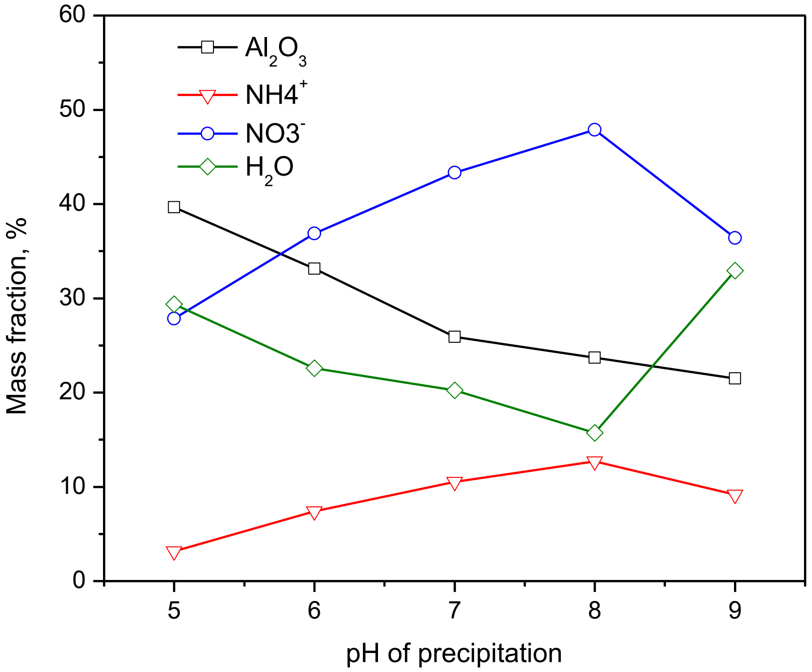

Figure S6. Composition of xerogels obtained at different pH values.

Figure S6 shows the content of components in xerogels at different CDJP process pH. It was shown that the ammonium and nitrate ion mass fraction increases with CDJP process pH growth. Interestingly, the amount of ammonium ions is significantly less than the number of nitrate ions in all xerogels.

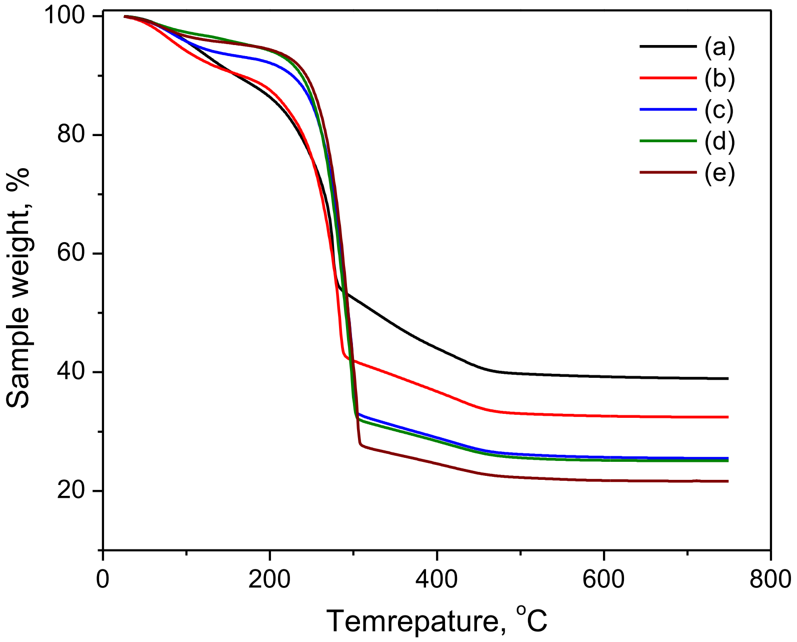

Figure S7. Thermal analysis of the xerogels: (a) Al-5; (b) Al-6; (c) Al-7; (d) Al-8 and € Al-9.

Figure S7 shows the weight loss curves for all xerogels samples. Three stages of decomposition were observed for all samples. In the first stage, the mass loss decreased, and the stage boundary expanded as the precipitation pH increased. The boundaries of the second stage were the same for all samples. However, the weight loss increased as the precipitation pH increased, with the trend remaining at the final stage of xerogels decomposition.

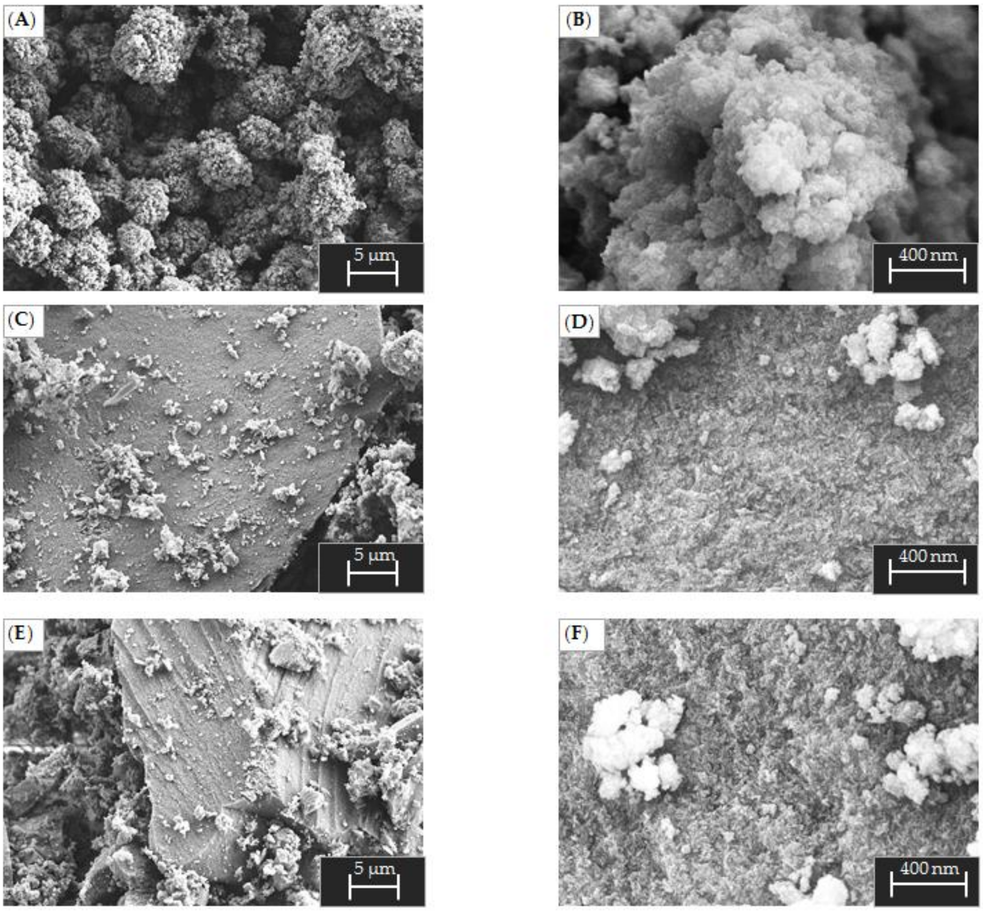

Figure S8. SEM-images of the samples Al-7, Al-8 and Al-9 after aging: (**A**) Al-7 2,5x; (**B**) Al-7 50x; (**C**) Al-8 2,5x; (**D**) Al-8 50x; (**E**) Al-9 2,5x and (**F**) Al-9 50x.

Figure S8 shows SEM images of Al-7, Al-8 and Al-9 samples after aging at 500 ^{O}C. The microstructure of Al-7 is similar to Al-5 and Al-6 and differs from Al-8 and Al-9, whose surface is densely agglomerated primary particles.

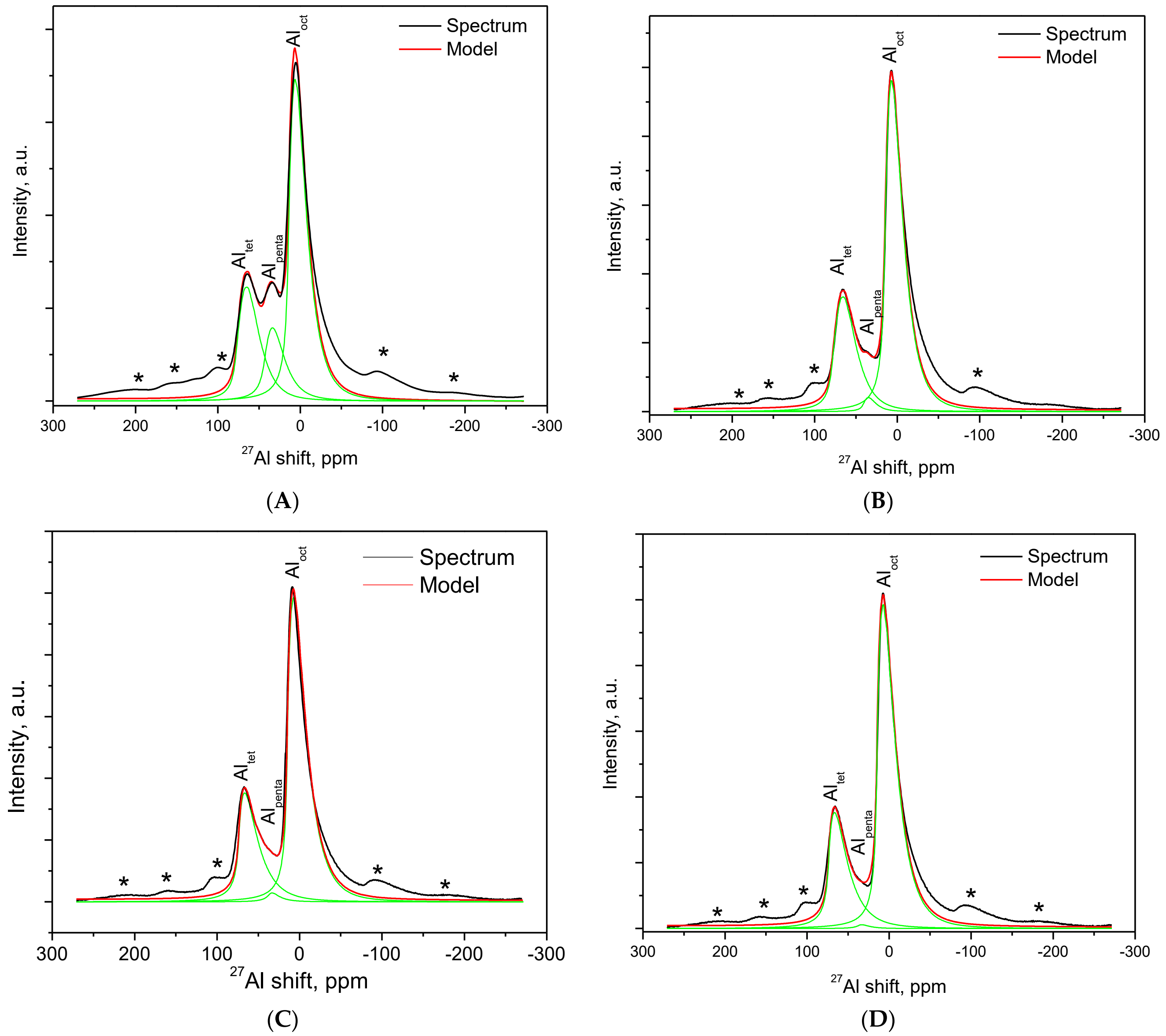

Figure S9. NMR spectra for samples after aging at 500 °C: (**A**) Al-6; (**B**) Al-7; (**C**) Al-8 and (**D**) Al-9.

As shown in Figure S9, Al-6 has the highest content of Al^{3+} penta-sites at 17 at.%. However, the fraction of penta-Al^{3+} decreases as the CDJP process pH grows to 2,24 at. %, 0,78 at. % and 0,34 at. % for Al-7, Al-8 and Al-9, respectively.
